# Sugar labeling information and online marketing strategies for hand-shaken tea drinks in northern Taiwan

**DOI:** 10.3389/fnut.2023.1273713

**Published:** 2023-11-14

**Authors:** Chi-Hsuan Liu, Te-Chih Wong, Mei Chung, Chyi-Huey Bai, Yi-Chun Chen

**Affiliations:** ^1^School of Nutrition and Health Sciences, Taipei Medical University, Taipei, Taiwan; ^2^Department of Nutrition and Health Sciences, Chinese Culture University, Taipei, Taiwan; ^3^Friedman School of Nutrition Science and Policy, Tufts University, Medford, MA, United States; ^4^Department of Public Health, School of Medicine, College of Medicine, Taipei Medical University, Taipei, Taiwan; ^5^School of Public Health, College of Public Health, Taipei Medical University, Taipei, Taiwan; ^6^Nutrition Research Center, Taipei Medical University Hospital, Taipei, Taiwan

**Keywords:** sugar-sweetened beverages, hand-shaken tea drinks, added sugar, labeling information, social media marketing, public health policy

## Abstract

**Background:**

Sugar-sweetened beverages (SSBs) are the main cause of excessive sugar intake and increased health risks. Food companies usually use social media to market SSBs in order to increase consumers’ purchase intentions. To reduce excessive added sugar consumption from hand-shaken tea drinks, Taiwan has implemented a mandatory policy requiring clear sugar content labeling. This study aimed to investigate the sugar label information and online marketing strategies for hand-shaken tea drinks in northern Taiwan.

**Methods:**

In this cross-sectional study, content analysis was employed to investigate the sugar labeling information and the current situation of online marketing in hand-shaken tea drink brands based in northern Taiwan. Seventy-two hand-shaken tea drink brands’ stores were visited to record their sugar labeling presentation methods, with brands lacking labeling, presenting incomplete labeling, or not offering customized sugar levels being excluded, resulting in 60 brands being chosen for the subsequent data collection process. The sugar and energy contents in 1,581 hand-shaken tea drinks were recorded and calculated. Subsequently, the sugar contents were assessed in accordance with World Health Organization (WHO) sugar recommendations (25 g/day), warning label criteria, and Taiwan’s regulations for low-sugar packaged beverages. Seven brands that had high online impressions were further selected and their marketing strategies in 560 Facebook posts were analyzed.

**Results:**

The presentation methods of labeling varied among the 60 brands, and only 42 brands had obvious and easily accessible labeling. The most common labeling presentation method was posters (*n* = 28). After converting the sugar content of half-sugar and low-sugar hand-shaken tea drinks, it was found that 60.2% of half-sugar beverages and 13.0% of low-sugar beverages exceeded 25 g of sugar per cup. Over 90% of brands had Facebook and Instagram accounts. The top marketing strategies for tea drink brands on Facebook were specific beverage information, brand information, and nutrition and health marketing. Most posts promoted sugar-sweetened beverages.

**Conclusion:**

Not all hand-shaken tea drink brands in this study followed Taiwan’s labeling regulations. Moreover, high sugar contents in hand-shaken tea drinks labeled as half-sugar and low-sugar could potentially lead people to unconsciously consume excessive amounts of sugar. Future research should explore the impact of online marketing strategies on SSBs consumption behavior and ways to mitigate it among the Taiwanese public.

## 1. Introduction

The World Health Organization (WHO) reports that excessive sugar intake, particularly in the form of sugar-sweetened beverages (SSBs), contributes to various health risks, including obesity, dental caries, and chronic diseases (1). Therefore, the WHO recommends that daily sugar intake should not exceed 10% of the total daily energy intake, and additional health benefits may be attained if this is further reduced to 5%. The WHO also announced that every country should develop effective health policies to reduce the intake of sugar and sugary drinks (1).

According to the 2013–2016 Nutrition and Health Survey in Taiwan, over 30% of Taiwanese people consume SSBs at least once per day, with a preference for tea-based beverages, such as sweetened tea and milk tea (2). Hand-shaken tea drink, also known as bubble tea or pearl milk tea, is a beverage that originated in Taiwan in the early 1980s (3). Typically, hand-shaken tea drinks involve tea-based and consist of tapioca (or other ingredients, such as jelly, pudding or fruits), ice and added sugar, and often include fruit juice or milk, all shaken together (4, 5).

The beverage consumption habits of Taiwan high school students Survey found that more than 60% of high school students frequently select hand-shaken tea drinks as their preferred SSBs choice, emphasizing the noteworthy role played by these drinks in the SSBs consumption patterns among Taiwanese youth (6). To discourage excessive sugar consumption from hand-shaken tea drinks, Taiwan has implemented a mandatory policy that necessitates the disclosure of sugar content through clear hand-shaken drink labeling. This disclosure shall be clearly stated in Chinese, demonstrated in forms of cards, menu notes, markings (labels) or notice boards, and put in places where consumers can easily notice it (see Supplementary material, for more details about the policy) (7).

Currently, many countries have already implemented regulations regarding nutritional labeling on packaged food products, as we known, Taiwan is currently the only country to regulate the labeling of energy and sugar content for hand-shaken tea drinks (8). Therefore, this study has provided insights into the labeling and sugar contents of hand-shaken tea drinks in Taiwan.

In recent years, the use of the Internet and digital media has rapidly grown. The 2020 Taiwan Internet Report revealed the growing prevalence of online food and beverage ordering and marketing in Taiwan. The report also drew attention to the expanding usage of LINE Pay, a digital payment service provided by LINE (9). LINE is a versatile communication application originating from Japan, offering messaging, voice/video calls, digital payments, and more, and it is predominantly used as the primary communication app in Taiwan. According to the “Digital 2022: TAIWAN” report, social media usage rate in Taiwan was 89.4%, with LINE (95.7%), Facebook (90.8%), and Instagram (70.6%) being the most frequently used social media platforms (10). Social media could be a critical factor contributing to dietary behaviors. Previous studies demonstrated that those who were more active online were more likely to be exposed to food marketing, thereby potentially increasing their consumption of unhealthy foods (11, 12). The 2022 Taiwan Internet Report indicated that 84.3% of people aged 18 years and older in Taiwan had used the Internet in the past 3 months. Furthermore, among the age group of 18 to 29, Internet usage stands impressively high at 99.4% (13). Being the main users of multiple social media platforms, Taiwanese people are easily exposed to the online marketing of several SSBs, possibly increasing their SSBs consumption.

Despite high SSBs consumption rates and greater exposure to online SSBs marketing among Taiwanese, there are few studies on hand-shaken tea drinks and social media marketing in Taiwan. Therefore, this study aimed to investigate the labeling information and online marketing strategies of hand-shaken tea drinks in Taiwan, as well as to examine the sugar contents of hand-shaken tea drinks. Additionally, we will evaluate compliance with the existing labeling laws and analyze how labeling practices align with consumption guidelines.

## 2. Materials and methods

### 2.1. Study design and procedures

**Figure 1:**
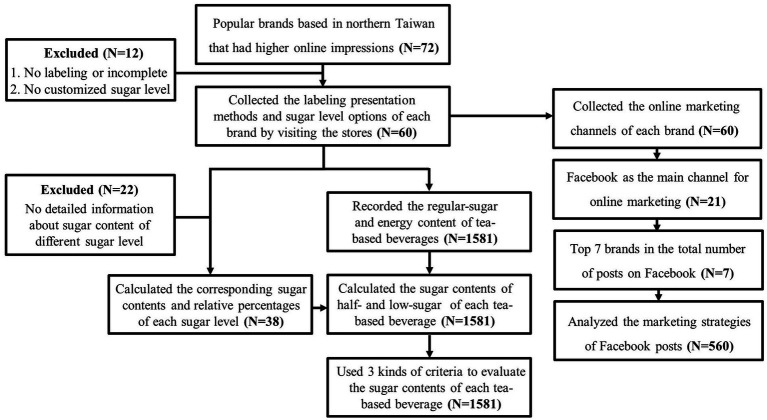
Hand-shaken tea drink brand search flow diagram.

In this cross-sectional study, content analysis was employed to investigate the labeling information of hand-shaken tea drinks and the current situation of online marketing. It was conducted in three main phases. Firstly, the labeling presentation methods and sugar level options for each hand-shaken tea drink brand were recorded by visiting stores. Secondly, information regarding the sugar and energy contents of various hand-shaken tea drinks offered by these brands was obtained from the labeling. Finally, the online marketing situation of each brand was investigated. The research process is illustrated in Figure 1.

According to Neuendorf’s conceptual framework (14), the researchers discussed and established three codebooks, namely: (a) labeling information and sugar level options among brands; (b) sugar and energy information for hand-shaken tea drinks; and (c) online marketing for hand-shaken tea drinks. A content analysis was used to transform the sugar labeling information and online marketing strategies into quantitative data for archiving and analysis. The first author instructed two researchers in the coding procedure, and duplicate coding was independently performed for reliability testing.

Content analysis is an important research method for objective, systematic, and quantitative descriptions of manifest communication content. It enables the transformation of large amounts of abstract, qualitative information into quantitative data (15). It is also both safe and cost-effective, as researchers can examine the nature and form of information contents, verify the accuracy of the data, and analyze them for patterns and trends (16). Since all the data used in this study were publicly available, ethical approval from the research committee was not required.

### 2.2. Sample selection and data collection

#### 2.2.1. Sugar labeling information of hand-shaken tea drink brands

Based on DailyView’s top 100 online impression ranking, 72 hand-shaken tea drink brands based in northern Taiwan were selected in this study. The brand list is reported in Supplementary Table S1. DailyView is a current affairs online big data analysis platform, which calculates the online impression these hand-shaken tea drink brands based on discussions among internet users, exposure on social media platforms, and the number of news articles within the past 3 months. A higher ranking indicates higher online impression.

We visited 72 hand-shaken tea drink brands’ stores, recording their sugar labeling presentation methods. The brands without labeling, or incomplete labeling, and did not offer customized sugar levels were excluded. Finally, 60 brands were chosen for the subsequent data collection process. Coded items included: (a) brand name; (b) labeling information; (c) sugar level options; and (d) corresponding sugar contents of common sugar levels (Supplementary Table S2).

Labeling presentation methods included: (a) menu notes; (b) at the order counter; (c) poster; (d) notice board; (e) on the back of the menu; (f) booklet; and (g) official website or online channels. Labeling acquisition included: (a) obvious labeling: customers can see the labeling before ordering without any assistance; (b) unobvious labeling: labeling information is accessible only upon customer inquiry; and (c) provided online after request: labeling information is only provided online.

**Figure 2:**
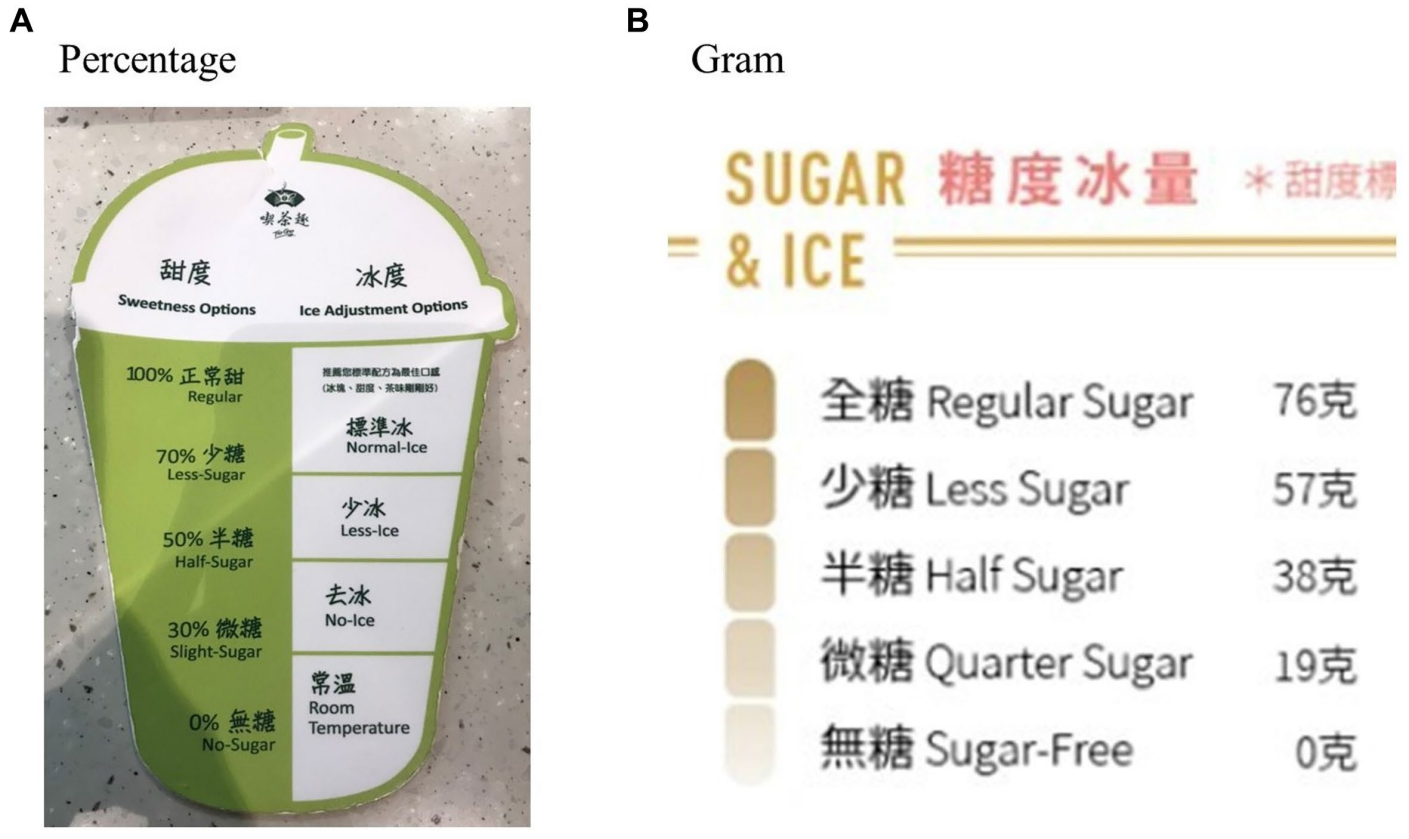
Examples of sugar level presentations **(A,B)**.

We recorded sugar level options in 60 brands. After excluding the brands without detailed information about sugar contents, the corresponding sugar contents and relative percentages of four common sugar levels (regular-sugar, less-sugar, half-sugar, and low-sugar) were calculated in 38 brands. There were two ways to present corresponding sugar contents of each sugar level: percentage (Figure 2A): marked as regular-sugar (100%) and the percentage of each sugar level. Considering that the composition of unflavored tea is relatively simple, and black tea is a common item in all brands, this study used the regular-sugar contents of a large (L) size cup of black tea as the base, then calculated the sugar contents of common sugar levels according to the percentage provided by the store; and grams (Figure 2B): grams of sugar in each sugar level. We directly recorded values from the labeling provided by stores.

#### 2.2.2. Sugar and energy information for hand-shaken tea drinks

According to the 2013–2016 Nutrition and Health Survey in Taiwan, the most commonly consumed type of beverage among Taiwanese was tea-based beverages (2). In this study, hand-shaken tea drinks were classified into two major categories and six subcategories: (a) hand-shaken tea drinks without toppings, comprising tea, milk tea, and fruit tea; and (b) hand-shaken tea drinks with toppings, comprising tea with toppings, milk tea with toppings, and fruit tea with toppings (Table 1). A total of 1,581 hand-shaken tea drinks were collected, coded items included (a) beverage name; (b) beverage category; (c) portion size; (d) energy content; and (e) sugar content at regular-, half-, and low-sugar levels (Supplementary Table S3).

**Table 1:** Definition and examples of each hand-shaken tea drinks.

Category	Definition and examples
Hand-shaken tea drinks	Beverages with a base made by steeping tea leaves in freshly boiled water.
Tea	Black tea, green tea, oolong tea, etc.
Milk tea	Black milk tea, green milk tea, oolong milk tea, etc.
Fruit tea	Passion fruit green tea, lemon green tea, kumquat lemon tea, etc.
Hand-shaken tea drinks with toppings	Hand-shaken tea drinks with pearls, pudding, coconut jelly, etc.
Tea with toppings	Black tea w/pearl, green tea w/pearl, black tea w/coconut jelly, etc.
Milk tea with toppings	Pearl milk tea, pudding milk tea, coconut jelly milk tea, etc.
Fruit tea with toppings	Passion fruit green tea w/pearl, grapefruit tea w/coconut jelly, peach black tea w/pearl, etc.

The regular-sugar contents of each beverage were obtained from the labeling provided by stores, half- and low-sugar contents of each beverage were calculated through the relative percentages. We used three kinds of criteria to evaluate if the sugar contents were high in 1581 hand-shaken tea drinks: (a) the WHO recommendation (>25 g/L cup): the WHO recommends that further health benefits would be attained if limiting free sugar intake to 25 g/day (1); (b) warning label criteria (>5 g/100 mL): the Chilean warning label & nutri-grade criteria for high-sugar beverages (17, 18); and (c) Taiwan’s regulations for low-sugar packaged beverages (>2.5 g/100 mL): according to “Regulations on Nutrition Labeling for Prepackaged Food Products” in Taiwan, sugar contents in low-sugar packaged beverages should not exceed 2.5 g/100 mL (19).

#### 2.2.3. Online marketing for hand-shaken tea drinks

In order to investigate the online marketing situation of 60 hand-shaken tea drink brands, we developed a codebook based on online marketing-related research and Internet reports in Taiwan (9, 10, 13, 20, 21). Coded items included: (a) brand information; (b) online platforms; (c) online ordering systems; (d) information in Facebook posts; and (e) Facebook marketing strategies (Supplementary Table S4). Online platforms included Facebook, LINE, Instagram, the official brand website, and other online marketing platforms. The online ordering systems included the brand’s online ordering services (Messenger, LINE, Nidin, and iCHEF) and food delivery platforms (UberEats, Foodpanda, and Cutaway).

Seven brands—DaYung’s, CoCo, Presotea, MACU, TEATOP, TRUEWIN, and KEBUKE—each with a high online impression and predominantly utilizing Facebook as their primary online marketing channel, regularly updating their fan pages at least once a week, and maintaining a higher total number of posts over the past 3 months, were further selected for an analysis of their Facebook marketing strategies. In total, 560 Facebook posts were collected from 7 brands, marketing strategies included: (a) co-branding; (b) special offers; (c) interactions with social media users, (d) specific beverage information; (e) cross-selling; (f) brand information; and (g) nutrient and health marketing.

### 2.3. Coding reliability and data analyses

We used kappa statistical tests to measure the inter-rater reliability of the two coders. Kappa coefficients of 0.61–0.80 indicated substantial agreement between the coders, while 0.81–1.00 indicated almost perfect agreement (22). In this study, 40 samples were first selected by two researchers to code and compare the results, and when kappa coefficients were <0.8, the coding discrepancies were discussed and resolved. Final kappa coefficients were all >0.80, indicating that the coded data of this study were quite reliable.

All statistical analyses were performed in IBM SPSS version 19 (23). The Kolmogorov–Smirnov test was used to examine whether the energy and sugar contents were normally distributed. Descriptive statistics used frequencies (*n*), percentages (%), and mean (range) for normally distributed data, while the median (interquartile range, IQR) was used for non-normally distributed data. The Kruskal–Wallis test was used to compare energy and sugar contents in different beverage categories. Two-sided *p*-values of <0.05 were considered statistically significant for all data analyses.

## 3. Results

### 3.1. Sugar labeling information of hand-shaken tea drink brands

#### 3.1.1. Labeling presentation methods

Among the 72 hand-shaken tea drink brands, seven brands had no labeling or incomplete labeling, and an additional five brands did not offer the option to customize sugar levels. Consequently, these 12 brands were excluded from the subsequent data collection process.

**Figure 3:**
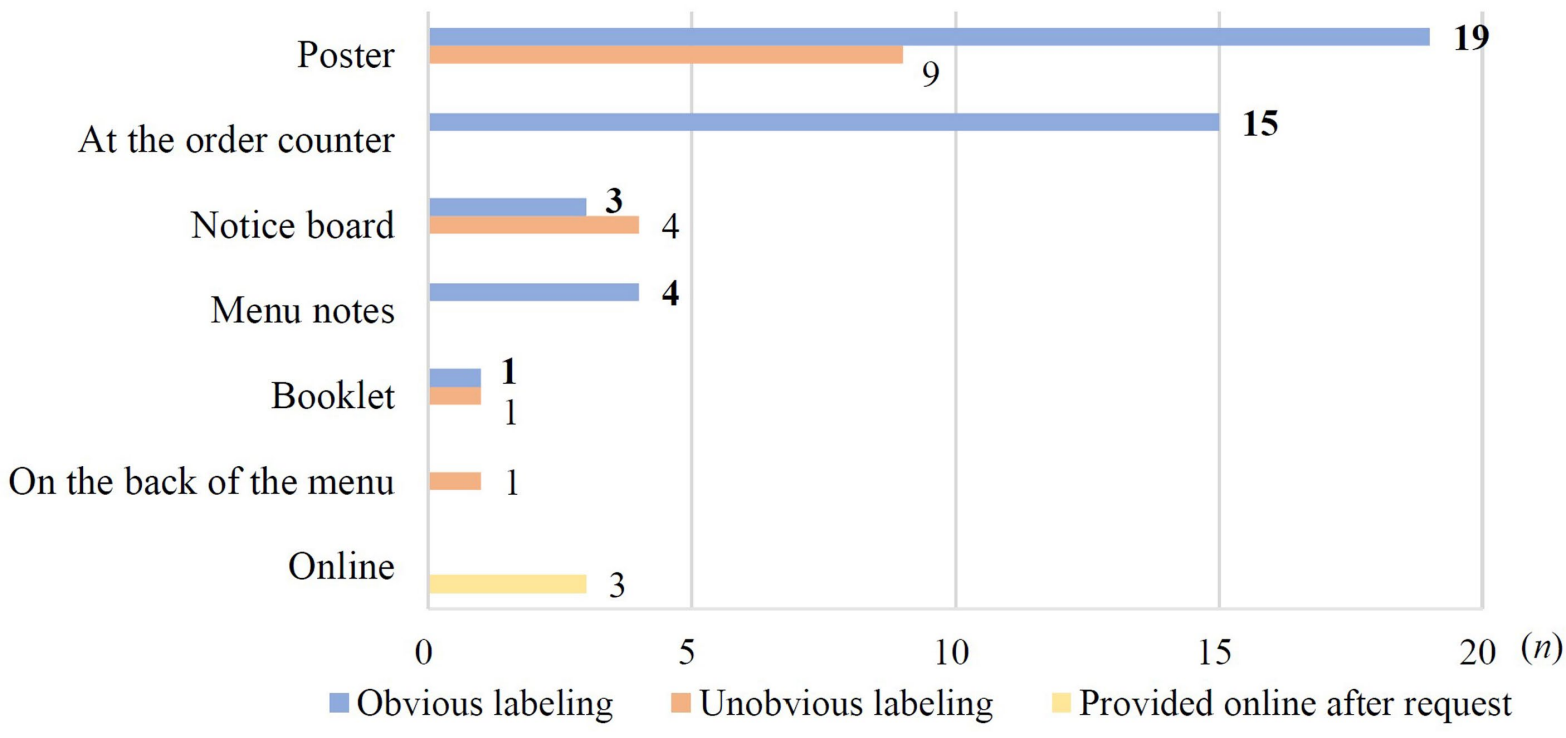
Distributions of labeling presentation methods.

The presentation methods of labeling varied among the 60 brands, the two most common methods were posters (*n* = 28; 46.7%) and labeled at the order counter (*n* = 15; 25.0%) (Figure 3). Only 42 brands (70.0%) had obvious and easily accessible labeling, like at the order counter (*n* = 15), menu notes (*n* = 4), some posters and notice boards. In certain brands, the labeling was unobvious, leading consumers to seek assistance in order to access the labeling information (*n* = 15; 25%). Furthermore, three brands (5.0%) provided sugar content information exclusively online.

#### 3.1.2. Sugar levels and corresponding sugar contents

Among the 60 brands, there were a total of 8 different sugar levels options: more sugar (*n* = 3), regular-sugar (*n* = 60), 90% sugar (*n* = 1), less-sugar (*n* = 50), half-sugar (*n* = 60), low-sugar (*n* = 60), very low sugar (*n* = 20), and sugar-free (*n* = 60). Each brand offered a range of sugar level options from three to seven kinds. However, only 38 brands provide detailed information regarding the corresponding sugar content for each sugar level. The remaining 22 brands only offer sugar level options without providing explanatory information about the sugar content for each sugar level.

The most of sugar content used percentages (*n* = 34) rather than grams (*n* = 4). Table 2 demonstrates significant variations in the sugar content or relative percentages across different brands with varying sugar levels. The relative percentage for less-sugar ranged from 70.0% to 81.8%, while the relative percentage for low-sugar ranged from 25.0% to 36.4%. The half-sugar variant exhibited a percentage range of 50.0% to 72.7%. This is due to a certain brand having a regular-sugar content of 50 c.c. of cane sugar syrup, while the half-sugar content is 40 c.c. Additionally, the maximum sugar content for some common sugar levels (less-sugar: 65.0 g/L cup, half-sugar: 45.5 g/L cup, and low-sugar: 27.3 g/L cup) exceeded the minimum sugar content found in regular-sugar (22.0 g/L cup) (Table 2).

**Table 2:** Sugar contents and relative percentages of common sugar levels.

	*N*	Per L Cup^a^	
		Sugar content (g)^b^	% of regular-sugar
Regular-sugar	38	51.1 (22.0–91.0)	100.0
Less-sugar	33	38.9 (15.4–65.0)	73.7 (70.0–81.8)
Half-sugar	38	25.8 (11.0–45.5)	50.6 (50.0–72.7)
Low-sugar	37	15.3 (6.6–27.3)	30.0 (25.0–36.4)
Sugar-free	38	—	—

a Large (L) cup = 600–750 mL.

b Data are presented as the mean (range).

### 3.2. Sugar and energy information for hand-shaken tea drinks

#### 3.2.1. Sugar and energy contents in hand-shaken tea drinks

In total, 1,581 hand-shaken tea drinks with a large cup size were collected. There were six different portion sizes: 600 mL (*n* = 72), 640 mL (*n* = 53), 650 mL (*n* = 86), 660 mL (*n* = 771), 700 mL (*n* = 505), and 750 mL (*n* = 94). The majority of 1,581 beverages were hand-shaken tea drinks (*n* = 1,234; 78.1%), including tea (*n* = 428; 27.1%), milk tea (*n* = 470; 29.7%), and fruit tea (*n* = 336; 21.3%).

Table 3 shows the sugar and energy contents in different hand-shaken tea drinks. The median sugar and energy contents were 53.6 g/L cup (8.0 g/100 mL) and 330.2 kcal/L cup (48.8 kcal/100 mL), respectively. Fruit tea (63.0 g/L cup) and fruit tea with toppings (79.0 g/L cup) had the highest sugar contents in two major categories, while milk tea (364.5 kcal/L cup) and milk tea with toppings (539.4 kcal/L cup) had the highest energy contents (Table 3).

**Table 3:** Sugar and energy contents of hand-shaken tea drinks in different categories.^a^

Category	*N* (%)	Sugar content (g)^b^		Energy content (kcal)^b^	
		Per cup^c^	Per 100 mL	Per cup^c^	Per 100 mL
Total	1,581 (100.0)	53.6 (43.0–70.0)	8.0 (6.4–10.3)	330.2 (231.0–451.2)	48.8 (34.9–66.5)
Hand-shaken tea drinks	1,234 (78.1)				
Tea	428 (27.1)	47.0^c^ (40.0–61.8)	7.1^c^ (5.8–9.1)	201.0^c^ (171.0–264.0)	30.3^c^ (25.0–40.0)
Milk tea	470 (29.7)	52.0^b^ (44.0–62.9)	7.9^b^ (6.5–9.1)	364.5^a^ (296.0–465.0)	53.6^a^ (44.7–70.5)
Fruit tea	336 (21.3)	63.0^a^ (50.0–79.0)	9.5^a^ (7.3–11.7)	302.2^b^ (246.0–366.2)	44.7^b^ (36.0–55.1)
*p*-value^d^		<0.001^***^	<0.001^***^	<0.001^***^	<0.001^***^
Hand-shaken tea drinks with toppings	347 (21.9)				
Tea with toppings	39 (2.5)	53.0^b^ (45.0–69.5)	7.6^b^ (6.8–10.4)	361.0^b^ (338.0–424.0)	53.7^b^ (48.4–60.6)
Milk tea with toppings	260 (16.4)	57.0^b^ (45.0–72.0)	8.6^b^ (6.6–10.7)	539.4^a^ (452.3–640.8)	79.9^a^ (67.2–93.4)
Fruit tea with toppings	48 (3.0)	79.0^a^ (64.0–97.0)	11.5^a^ (9.1–14.7)	387.5^b^ (306.5–473.6)	57.2^b^ (44.0–67.3)
*p*-value^d^		<0.001^***^	<0.001^***^	<0.001^***^	<0.001^***^

a Data are presented as the median (inter-quartile range).

b Each column a > b > c.

c L cup = 600–750 mL.

d ^***^p < 0.001, obtained using the Kruskal–Wallis with Dunn’s test.

#### 3.2.2. High sugar rates with different criteria

**Figure 4:**
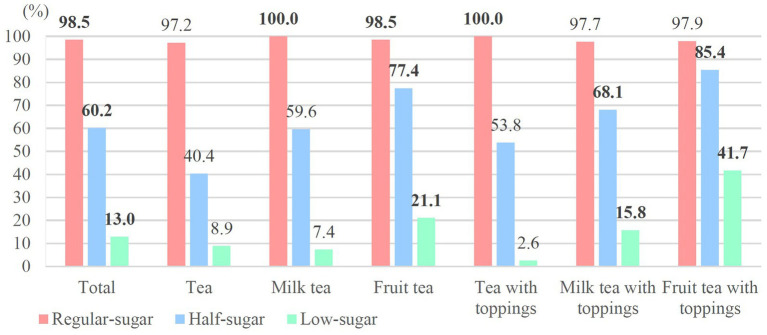
High sugar rates according to WHO recommendations.

**Figure 5:**
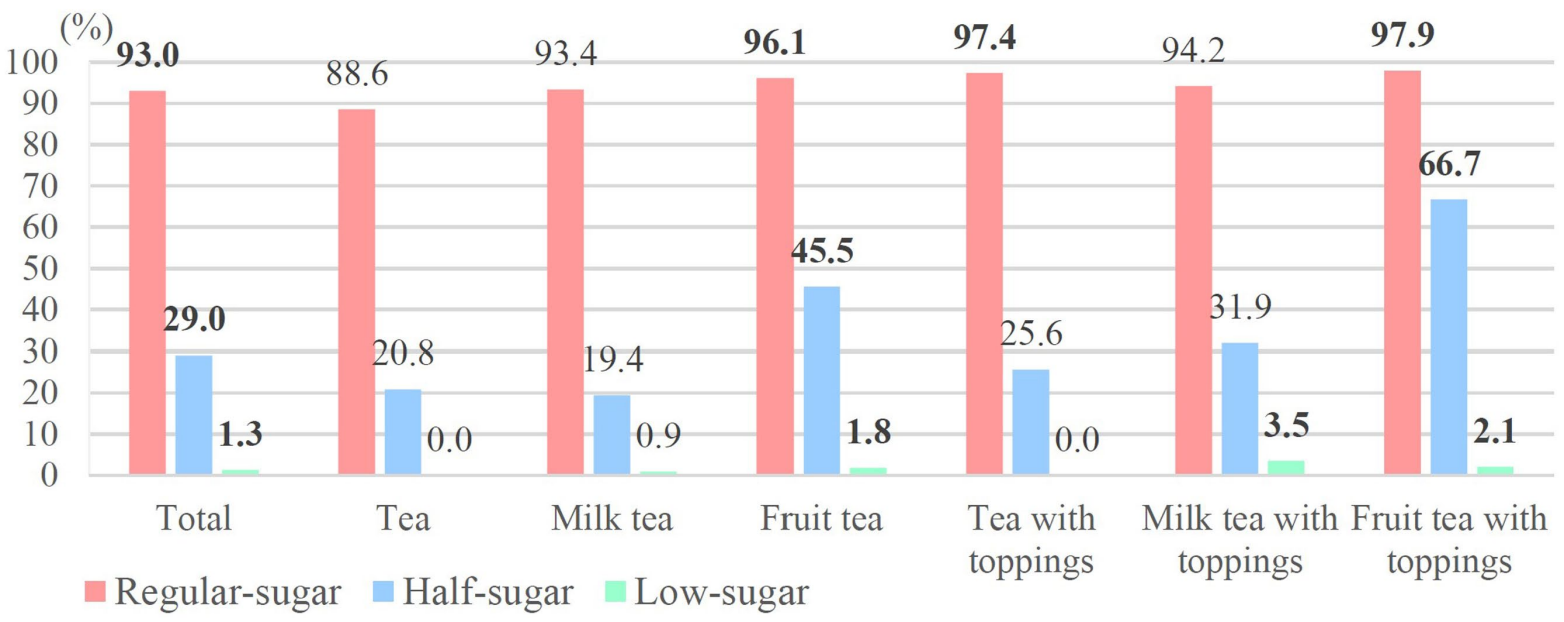
High sugar rates according to warning label criteria.

Figures 4, 5 display high sugar rates based on WHO recommendation and warning label criteria in each category of beverages at regular-sugar, half-sugar, and low-sugar levels. In accordance with WHO recommendation (>25 g/L cup), 98.5% of the regular-sugar beverages contained more than 25 g sugar/L cup; both milk tea and tea with toppings exceeded the standard across all items. About 60% of half-sugar beverages did not meet the criteria, with higher proportions of fruit tea with toppings (85.4%), fruit tea (77.4%), and milk tea with toppings (68.1%) exceeding the standard. The categories of low-sugar beverages with higher high sugar rates were still fruit tea with toppings (41.7%), fruit tea (21.1%), and milk tea with toppings (15.8%) (Figure 4).

High sugar rates decreased in warning label criteria (>5 g/100 mL) compared to the WHO recommendation, with 93% of the regular-sugar beverages being classified as high sugar; fruit tea with toppings (97.9%), tea with toppings (97.4%) and fruit tea (96.1%) had higher high sugar rates. Further, 66.7% of fruit tea with toppings and 45.5% of fruit tea had high sugar contents at the half-sugar level. Twenty high sugar beverages at the low-sugar level were milk tea with toppings (*n* = 9), fruit tea (*n* = 6), milk tea (*n* = 4), and fruit tea with toppings (*n* = 1) (Figure 5).

**Figure 6:**
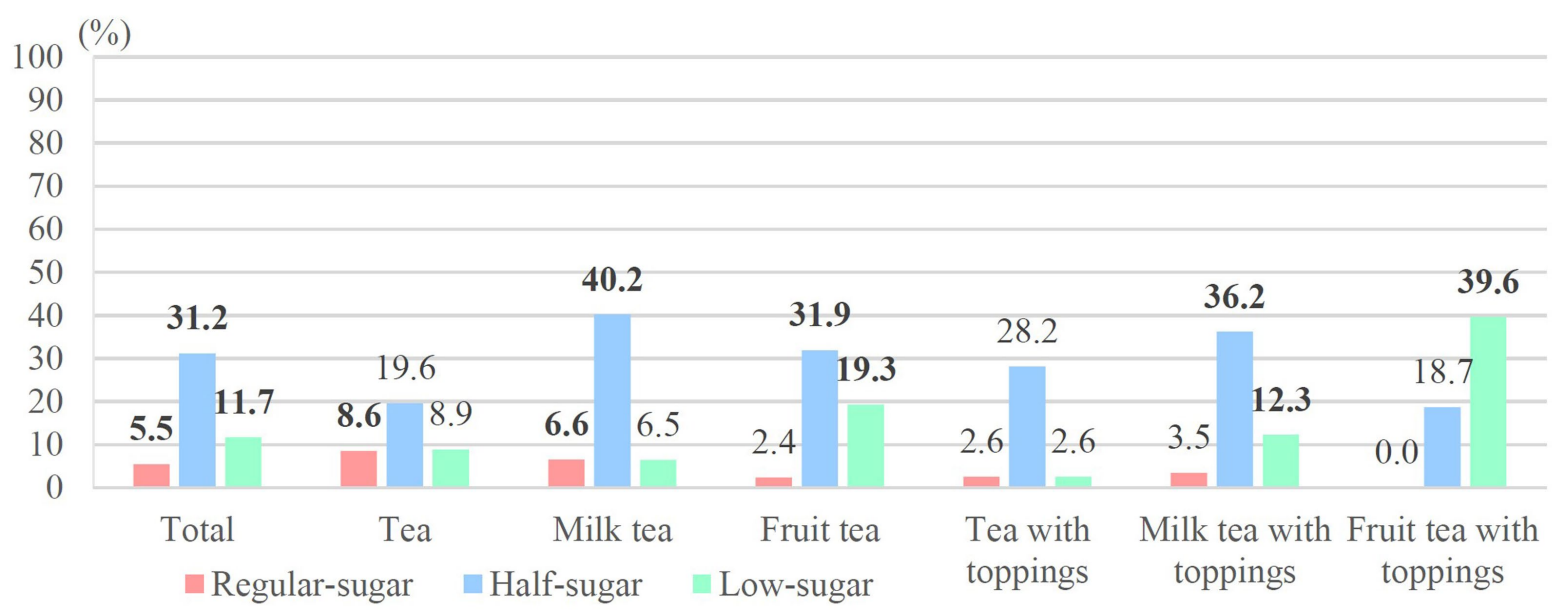
Distributions of >25 g sugar per large (L) cup that were “not” classified as being high sugar.

**Figure 7:**
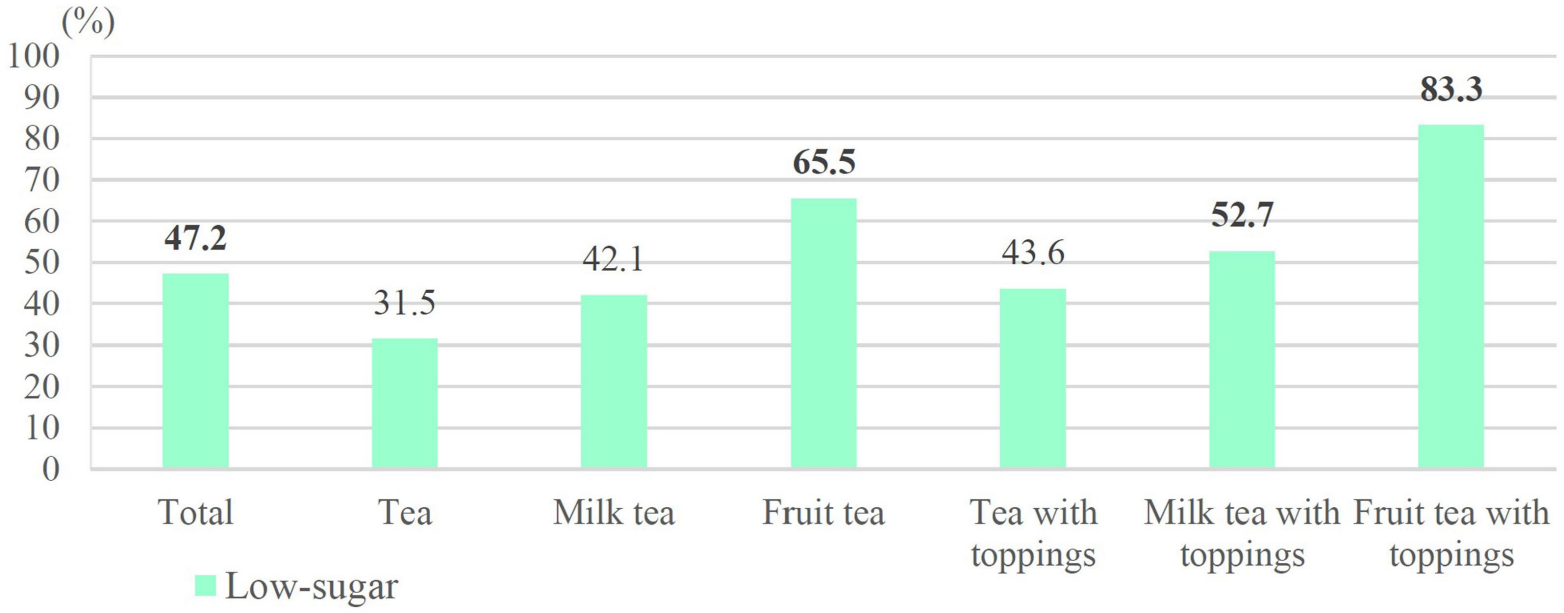
Excessive sugar rates according to Taiwan’s regulations (low-sugar beverages).

Figure 6 shows the distributions of hand-shaken tea drinks with a sugar content of >25 g/L cup but a sugar concentration of <5 g/100 mL. Proportions of half-sugar beverages (31.2%) were higher than regular- and low-sugar beverages (5.5% and 11.7%, respectively), especially for milk tea (40.2%), milk tea with toppings (36.2%), and fruit tea (31.9%); among low-sugar beverages, fruit tea with toppings (39.6%) and fruit tea (19.3%) exhibited higher percentages than other beverage categories. According to Taiwan’s regulations for low-sugar packaged beverages (>2.5 g/100 mL), almost half of the low-sugar beverages exceeded the standard. The excessive rates of each category from high to low were as follows: fruit tea with toppings (83.3%), fruit tea (65.5%), milk tea with toppings (52.7%), tea with toppings (43.6%), milk tea (42.1%), and tea (31.5%) (Figure 7).

### 3.3. Online Marketing for hand-shaken tea drinks

#### 3.3.1. Online platforms and online ordering systems

All brands had official Facebook accounts, and over 90% of the brands had official Instagram accounts (*n* = 57; 95.0%). More than 75% of the brands had official websites (*n* = 46; 76.7%), and 28 brands had official LINE accounts (46.7%). One brand had other online marketing channels such as YouTube and TikTok. All brands collaborated with food delivery platforms, and 60% of the brands offered their own online ordering services (*n* = 36).

#### 3.3.2. Marketing strategies on Facebook

**Figure 8:**
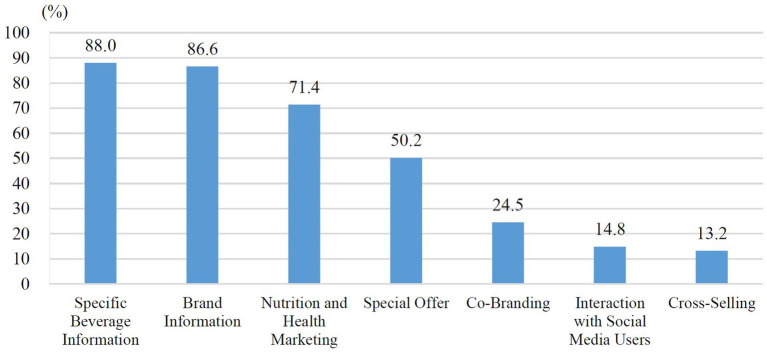
Online marketing strategies of hand-shaken tea drink brands.

Based on online impressions, seven hand-shaken tea drink brands were further selected for analysis of their marketing strategies on Facebook: DaYung’s, CoCo, Presotea, MACU, TEATOP, TRUEWIN, and KEBUKE. We took screenshots of their Facebook posts from September to December 2021, ultimately collecting 560 posts in total. The top three major strategies were specific beverage information (*n* = 493; 88.0%), brand information (*n* = 485; 86.6%), and nutrition and health marketing (*n* = 400; 71.4%) (Figure 8). Specific beverage images were the most common subcategory strategy in the posts (*n* = 460; 82.1%), followed by the URL of the brand’s official website or brand-related news articles (*n* = 388; 69.3%) and those mainly promoted fruit-based beverages (*n* = 284; 50.7%) (Supplementary Table S5).

Among 493 posts containing specific beverage information, only 8 (1.6%) of them encouraged consumers to choose or promoted sugar-free beverages. Out of 400 nutritional and health marketing posts, only 8 marketed sugar-free beverages, 375 (93.8%) marketed sugary drinks, and 17 marketed beverage ingredients.

## 4. Discussion

### 4.1. Labeling presentation methods in stores

The WHO endorses that nutrition labeling can help people choose healthier meals, leading to reductions in the overall intake of energy, sugar, sodium, and fat (24). In addition to nutrition labeling on packaged foods, nutrition labeling for freshly prepared meals or beverages is also crucial in promoting healthy eating. Taiwan’s hand-shaken tea drinks have flourished in recent years; hence the Ministry of Health and Welfare enacted regulations on the labeling of hand-shaken tea drinks, requiring beverage chains to provide labeling on site in 2015 (7). As we known, Taiwan is currently the only country that has mandated the disclosure of sugar contents in hand-shaken tea drinks (8). This study included 72 hand-shaken tea drink brands, and nearly 10% of them did not follow the regulation, only 60 brands had complete labeling of beverages, but they were presented in a variety of ways. Furthermore, it was found that the labeling presentation methods of some brands are not conducive to the public’s acquisition. Instead, they require consumers to actively seek out the labeling information by requesting it or relying on online sources. This finding indicates that despite the implementation of a mandatory policy for labeling hand-shaken tea drinks in Taiwan, the existing regulations lack clarity and comprehensiveness, such as the meaning of clear labeling and the format of labeling information. Although penalties are explicitly defined for non-compliance with the regulations, enforcement of the policy remains inadequate, resulting in confusion and ineffectiveness of the current hand-shaken tea drink labeling protocols (25).

There is a noticeable absence of research focused on hand-shaken tea drinks labeling. In contrast, a scoping review found that more than 10 countries legislated for voluntary or mandatory menu labeling in restaurants, providing menu nutrition labeling not only incentivizes restaurants to reformulate their recipes and create healthier menu options but also encourages consumers to choose healthier food options (8). Several countries, such as South Korea, Australia, and the United States, require energy labeling on the menu or menu boards in restaurants, to ensure that consumers can use the labeling information as a reference when ordering food (26–30). Given the resemblance in ordering dynamics between restaurants and hand-shaken tea drinks, relevant research pertaining to restaurant menu labeling could potentially provide valuable guidance for improving the labeling practices of hand-shaken tea drinks. An Australian study found that after the implementation of mandatory menu labeling in fast food restaurants, the energy contents of meals purchased by consumers had significantly decreased, indicating that menu labeling can enhance consumers’ understanding of meals, leading them to choose lower-energy meals (31). A US study found that after the implementation of menu labeling, a decrease in the level of 60 calories/transaction was observed among 104 restaurants (32). Another study that examined trends in the energy and macronutrient compositions of menu items from 2012 to 2018 found that energy and saturated-fat contents of newly introduced items had significantly declined (33). Those studies demonstrated that nutrition information should be provided where consumers can see it when ordering, such as next to the items’ name or price, in order to exactly guide people to choose a healthier diet, and at the same time motivate restaurants to change the ingredients of their meals and introduce healthier meals. However, only four brands in this study provided energy and sugar labeling on the menu. Further research could be undertaken to examine consumers’ perspectives on various methods of sugar content presentation or the effects of these diverse presentation methods on consumer behavior.

Our study found that more than 95% of hand-shaken tea drink brands had several online marketing channels, including Facebook, Instagram, official websites, LINE, YouTube, and TikTok, allowing consumers to access information on the latest promotions, popular beverages, store locations, etc. through different social media platforms. Moreover, all brands were cooperating with online delivery platforms, and nearly 60% of brands had their own online ordering systems, showing that online platforms are also an important channel for people to realize the brand and purchase beverages. However, the current policy only mandates provisions for labeling in stores, while online platforms may voluntarily provide labeling. Consumers might not be able to get nutrition information about beverages if they use online ordering systems. Canada has a mandatory policy for menu labeling which differs from those of other countries; it further mandates that online ordering systems should provide the same information as menu labeling; in this way, consumers can read the information when ordering online (34). According to the results of online platforms in this study, all hand-shaken tea drink brands have social media and online ordering systems, which means that mandatory labeling should not be limited to stores but should also extend to online platforms so that consumers can read the complete labeling information when ordering online.

In this study, each brand offered a range of three to seven sugar-level options, a total of 8 different sugar levels were collected, with the common sugar levels being regular-sugar, less-sugar, half-sugar, low-sugar, and sugar-free. Approximately 40% of the brands fail to provide clear information regarding the sugar content corresponding to different sugar levels, and the relative sugar content of common sugar levels varied across different brands. It was observed that some variants labeled as “less-sugar” had a sugar content of 80% of regular-sugar, while others had a sugar content of 70% of regular-sugar, and the “low-sugar” option had a sugar content of 30% of regular-sugar. Given the inconsistency between sugar levels and corresponding sugar content across different brands, coupled with inadequate labeling practices, consumers are prone to confusion and are more likely to make erroneous choices. Standardizing the relative sugar content of different sugar levels in hand-shaken tea drinks can facilitate consumer recognition and informed decision-making.

### 4.2. Portion sizes and sugar contents of hand-shaken tea drinks

We collected 1,581 hand-shaken tea drinks with a size L cup in this study, and there were six different portion sizes ranging from 600 to 750 mL, with 660 and 700 mL being the most common. The current regulations require that the actual capacity of each portion size should be written in the labeling, but there is a lack of clear rules or recommendations on how much capacity is appropriate for hand-shaken tea drinks. Taiwan’s Food Nutritional Database used 700 mL as the portion size when describing hand-shaken tea drinks (35). In Australia, 600 mL is used as the standard portion size on beverage labeling (36). These mean that 600–700 mL are typically consumed by most people in one portion.

In our study, the median sugar content of hand-shaken tea drinks was 9.5 g/100 mL for fruit tea, 7.9 g/100 mL for milk tea, and 7.1 g/100 mL for tea. These results were similar to previous studies of supermarket beverages in Taiwan (fruit/vegetable juices and beverages: 9.6 g/100 mL; milk tea: 7.5 g/100 mL) (37) and in China (fruit/vegetable juices and beverages: 10.0 g/100 mL; tea beverages: 7.0 g/100 mL) (38). For high sugar rates, 98.5% of regular-sugar beverages contained more than 25 g of sugar per L cup, and more than 90% of regular-sugar beverages were labeled as high-sugar according to warning label criteria (>5 g/100 mL) in this study. Previous studies found that 41.6% of Taiwan’s packaged SSBs contained more than 25 g of sugar, and nearly 70% of SSBs were high in sugar according to the Chilean warning label (39). Thus, while the sugar concentration of hand-shaken tea drinks was similar to that of packaged SSBs, the high sugar content of L cups (600–750 mL) of hand-shaken tea drinks was much more significant than that of packaged SSBs (250–350 mL). Our study also found that among hand-shaken tea drinks containing less than 5 g of sugar per 100 mL, there were still some that contained more than 25 g of sugar per L cup, especially at the half-sugar (31.2%) and low-sugar (11.7%) levels. The findings indicated that even in hand-shaken tea drinks with low-sugar concentrations, the overall sugar content can still be significantly high due to the large volume of the beverage. Consequently, if we were to assess the sugar content using the criteria set forth for warning labels, numerous beverages with substantial sugar content might be overlooked. There were 13% of low-sugar beverages that contained more than 25 g of sugar, and 47.2% of them exceeded the standard according to Taiwan’s regulations for low-sugar beverages (>2.5 g/100 mL). A high sugar content in a cup of beverage can also cause people to consume too much sugar, but the current regulations only regulate the claims of sugar for packaged beverages. Many people might believe that choosing half-sugar or low-sugar hand-shaken tea drinks would be a healthier option. However, this study revealed that a considerable proportion of half-sugar or low-sugar beverages still exceeded the WHO recommendation. Our government should re-examine and establish sugar content standards for different sugar levels of hand-shaken tea drinks, so that the industry can have a basis for sugar claims, and consumers can also use them as a standard to choose an appropriate sugar level for themselves. However, whether consumers will calculate the sugar content when buying tea drinks or understand the difference between the sugar content and the recommendation of sugar intake still needs to be further explored.

A previous study found that a larger portion size contributed to larger consumption by consumers and resulted in excessive energy intake, especially of high-calorie foods (40). Similarly, hand-shaken tea drinks with a much larger volume than packaged beverages, make it easier for consumers to increase their sugar intake. High sugar content in a cup of beverage can lead to excessive sugar intake from the beverage, which may increase health risks such as obesity, dental caries, and metabolic syndrome (41–43). The US FDA has updated the serving size standard on the nutrition facts label, making it more realistically reflect how much people typically eat at one time. The updated label makes it easier for consumers to read and make better-informed food choices (44). Taiwan should also re-examine and set appropriate standards for portion sizes and sugar contents for beverages based on current dietary habits, beverage types, and sugar contents to avoid excessive sugar intake by consumers.

### 4.3. Online marketing strategies

In our study, the majority of hand-shaken tea drink brands attempted to increase the beverage and brand exposure, and over 90% nutrition and health marketing was used to promote SSBs, especially those containing fruit. However, based on results of sugar contents in hand-shaken tea drinks, the highest sugar contents were found in fruit teas (fruit teas: 63 g/L cup; fruit teas with toppings: 79 g/L cup). Studies in Taiwan (37), China (38, 45), and New Zealand (46) also found similar results that juice and fruit drinks had the highest sugar contents among supermarket beverages. According to previous studies on perceptions of SSBs, participants usually thought that fruit was healthy, were not sure of the difference between fruit and fruit drinks, or were unaware that fruit drinks may also contain added sugar (38, 47, 48). The long-term excessive intake of fruit drinks can cause several health risks, such as weight gain, type 2 diabetes, and metabolic syndrome (49–51). If the industry continues to market fruit drinks with nutrition- and health-related claims, people may easily misinterpret health ideas about them. Therefore, it is necessary to raise people’s awareness of fruit drinks and also examine the truthfulness of marketing.

Previous studies on nutrition and health marketing in Taiwan found that 80.3% of packaged beverages with more than 25 g of sugar per portion had health marketing such as “fresh fruit and vegetable images” or “nutrition and ingredient claims” (52), and nearly half of children’s snacks and beverages had natural images such as fresh fruits and vegetables, and up to 95.8% of the products claiming “no added sugar” were high in sugar, containing more than 10% of total calories in sugar (53). In addition, half of commercial infant foods with health, ingredient, and nutritional claims were high in sugar, and 72.9% of products claiming “no added sugar” contained more than 10% of total calories in sugar (54). Foreign studies also found that food companies often use nutrition and health marketing to promote unhealthy products (55–58). This study found that fruit-related claims, including text and images, and words such as “fresh” were the most common health marketing used by hand-shaken tea drink brands. A study in Uruguay found similar results, with images of fresh fruits and vegetables being most common on food packaging, followed by claims such as “natural” and “fresh” (59). According to an online survey in Taiwan, 40.0% of participants thought products that claimed “no added sugar” were healthier, and more than half of them believed that products claiming “no added sugar” were more natural, healthier, and lower in sugar content (60). A Danish study also found that nutrition and health claims led consumers to believe that the product was healthier, especially when added to unhealthy products such as potato chips, pizzas, and cakes (61). A Belgian study on teenagers’ perceptions of health marketing found that teenagers did not think products with fruit images were healthier, but were more likely to buy them because they thought that kind of product tasted better (62).

Previous studies on Facebook food marketing have found that the main marketing techniques are mostly entertainment and engaging with social media users (20, 63, 64) Unlike the entertainment and interactive marketing often found in previous studies, health marketing often used by Taiwanese food industries, regardless of the actual ingredients of the product, makes people think that the product is natural and healthy, which in turn increases their willingness to buy it. In particular, images of fresh fruits and vegetables are likely one of the most important factors in increasing the consumption of SSBs among teenagers. However, there is still no policy to regulate online marketing. Most of the regulations on unhealthy food marketing that target children are limited to traditional media, such as prohibiting the marketing of unhealthy foods at certain times or on children’s TV channels, but there are no restrictions on unhealthy food marketing on general channels and online platforms (65–69).

In May 2022, at the 75th World Health Assembly (WHA), the WHO announced that governments should address the impacts of all forms of food marketing on children’s health and proposed the following three comprehensive policy approaches that are considered to have the highest potential to achieve the desired policy impacts: (a) eliminating all forms of marketing of foods that are “high in saturated fats, trans-fatty acids, free sugars, or salt” to which a broad range of children are exposed; (b) eliminating all forms of food marketing to which a broad range of children are exposed; and (c) eliminating all forms of marketing to which a broad range of children are exposed (70). In recent years, the UK, Spain, and Singapore have enacted regulations to extend marketing restrictions of unhealthy foods or beverages to online games, social media, outdoor advertising, and other channels, rather than being limited to traditional media (18, 71, 72), but the effectiveness of their implementation remains to be confirmed.

At present, marketing regulations on social media are still mostly set by self-regulatory organizations or the industry itself, with a lack of mandatory government regulations. Further research is need to investigate how current marketing strategies on social media affect SSBs consumption. We should develop health policies through the same channels to counteract online marketing, thereby directly or indirectly reducing the consumption of SSBs by individuals, or even by extension, for public health.

### 4.4. Limitations

This study has some limitations. First, our study was limited by a shortage of researchers, locations, and time, and only included hand-shaken tea drink brands based in northern Taiwan and L cups of hand-shaken tea drinks. However, there are also hand-shaken tea drink brands based in middle and southern Taiwan, as well as non-tea-based beverages, such as milk, smoothies, coffee, etc. Labeling information among different brands and sugar contents of different types of beverages would possibly have contributed to different results.

Second, we calculated the sugar contents of beverages with varying sugar levels using the sugar percentages provided by the stores and the sugar contents of regular-sugar beverages. It remains uncertain whether beverages with different sugar levels actually exceed the sugar criteria, as current regulations only require the labeling of sugar content for regular-sugar beverages. Third, we used total sugar rather than added sugar to evaluate sugar contents of hand-shaken tea drinks because their labeling only indicate the total sugar contents. Further research should be conducted to promote labeling policies on hand-shaken tea drinks. Finally, the information collected in this study was obtained from the labeling provided by the manufacturers without outside independent confirmation.

## 5. Conclusion

The study revealed that the sugar labeling of hand-shaken tea drinks in Taiwan is confusing and sometimes inaccessible due to inconsistent formatting, making it difficult for people to comprehend. Additionally, high sugar contents of hand-shaken tea drinks labeled as half-sugar and low-sugar may lead people to unconsciously consume excessive sugar. Further research was needed to explore consumer perceptions of different sugar level presentation methods and their impact, informing potential revisions to labeling regulations. Additionally, investigating the effects of online marketing strategies on sugar-sweetened beverage consumption could aid in developing relevant health policies to mitigate potential negative impacts on public.

## Data availability statement

The original contributions presented in the study are included in the article/Supplementary material, further inquiries can be directed to the corresponding author.

## Author contributions

C-HL: Data curation, Methodology, Writing – original draft. T-CW: Writing – review & editing, Data curation, Formal analysis. MC: Writing – review & editing, Validation. C-HB: Writing – review & editing, Formal analysis. Y-CC: Writing – review & editing, Project administration, Supervision.
