# Supplementary material for: Sugar labeling information and online marketing strategies for hand-shaken tea drinks in northern Taiwan
Source: Front Nutr. 2023 Nov 14;10:1273713. doi: 10.3389/fnut.2023.1273713 (PMC10682441; doi:10.3389/fnut.2023.1273713)
Supplement: Supplementary file 1 [file Data_Sheet_1.docx]

**Supplementary Material**

**Regulation for the Labeling of Freshly Made Beverages in Chain Drink Stores, Convenience Stores, and Fast Food Restaurants**

Amended on October 5, 2020

**Article 1**

The Regulations are prescribed in accordance with Paragraph 2 of Article 25 of the Act Governing Food Safety and Sanitation.

**Article 2**

Chain beverage stores, convenience stores and fast food restaurants (hereinafter referred to as chain stores) that have a taxation registration certificate and sell freshly made beverages shall follow this regulation.

**Article 3**

The chain store stated in Article 2 refers to a company or business registered under the same name, or using the same name by means of franchise, authorization, etc.

**Article 4**

Beverages freshly made shall be labeled total amount of sugar and calories. The range of allowable error of its label value should comply with the “Regulations on Nutrition Labeling for Prepackaged Food”. The total amount of sugar and calories may also be expressed by labeling the highest value. If the highest value is labeled, the term “highest value” shall be added.

The amount of sugar mentioned above may be converted to the number of cube sugars (5 grams per cube)

The labeling of product name for tea, coffee, fruit and vegetable beverage shall meet the following requirements

1. Tea beverages:
2. The country (place) of origin of the tea leaves. If the beverage contains tea leaves from more than 2 countries, the name of the beverage shall indicate the origin countries in the order according to the content from high to low.
3. The beverage that does not made from tea leaves but from additives such as tea essence shall add the words “○○ flavor” in its name.
4. Coffee beverages:
5. The country (place) of origin of the coffee material. If the beverage contains coffee materials from more than 2 countries, the name of the beverage shall indicate the origin countries in the order according to the content from high to low.
6. The highest value of the total caffeine content shall be labeled, and the term “highest value” shall be added. Alternatively red, yellow and green colors may be used to differentiate its caffeine content by labeling a symbol or a figure.
7. If caffeine content of a cup of coffee is more than 201 mg, it shall be labeled with color red.
8. If caffeine content of a cup of coffee is between 101 mg and 200 mg, it shall be labeled with color yellow.
9. If caffeine content of a cup of coffee is less than 100 mg, it shall be labeled with color green.
10. Fruit and vegetable beverages:
11. Only products contain more than 10% of fruit and vegetable juice, the name of “○○ juice” can be used.
12. If a product contain fruit and vegetable juice less than 10%, its product name shall be labeled as“○○ drink”, or other synonymous words.
13. A product that does not contain fruit and vegetable juice shall be labeled the words “○○ flavor” in its name.

**Article 5**

The labeling of this regulation shall be clearly stated in Chinese, demonstrated in forms of cards, menu notes, markings (labels) or notice boards, and put in places where consumers can easily notice it.

The character unit of the menu notes and markings (labels) mentioned in the preceding paragraph should not be smaller than 0.2 cm in length and width. The character unit of other means of labeling should not be smaller than 2 cm in length and width.

**Supplementary Table S1.** Hand-shaken Tea Drink Brands List.

| **No.** | **ID** | **Brand name** |  | **No.** | **ID** | **Brand name** |
| --- | --- | --- | --- | --- | --- | --- |
| 1 | 1001 | Huada Milk Tea |  | 37 | 1042 | Hanlin Tea House |
| 2 | 1002 | Cha Nung |  | 38 | 1043 | Little things in life |
| 3 | 1003 | TP TEA |  | 39 | 1044 | TAO TAO TEA |
| 4 | 1004 | Ten Ren Tea |  | 40 | 1046 | Le Phare |
| 5 | 1006 | TRiZZO |  | 41 | 1048 | DAY DAY DRINK |
| 6 | 1007 | DaYung's |  | 42 | 1049 | Tea top |
| 7 | 1008 | Milksha |  | 43 | 1050 | Wanpo Tea Shop |
| 8 | 1009 | Coco |  | 44 | 1053 | Pearl milk tea |
| 9 | 1011 | Gong cha |  | 45 | 1056 | Brian Black Tea |
| 10 | 1013 | Tiger Sugar |  | 46 | 1057 | THE ALLEY |
| 11 | 1014 | OPEN YOUR DOOR |  | 47 | 1058 | Hechalou Tea |
| 12 | 1015 | Presotea Tea |  | 48 | 1059 | Tredecimber |
| 13 | 1016 | Comebuy |  | 49 | 1060 | UNOCHA |
| 14 | 1018 | KEBUKE Tea |  | 50 | 1061 | Don't yell at me |
| 15 | 1019 | Chatime |  | 51 | 1062 | Oregin |
| 16 | 1021 | Chingshin |  | 52 | 1063 | Europa world tea house |
| 17 | 1022 | Yifang Taiwan Fruit Tea |  | 53 | 1065 | Bubble Lee |
| 18 | 1023 | CHUN YANG |  | 54 | 1066 | Guiji |
| 19 | 1024 | TRUEDAN |  | 55 | 1068 | John Tea Company |
| 20 | 1025 | LiCha Frucht |  | 56 | 1070 | Shang Yu Lin |
| 21 | 1026 | Spade Tea |  | 57 | 1071 | Nanhaisado |
| 22 | 1027 | CHAGE |  | 58 | 1072 | Black tea house |
| 23 | 1028 | Slowly, Yo |  | 59 | 1074 | A Nice Holiday |
| 24 | 1029 | Okla tea |  | 60 | 1076 | Nine Stream |
| 25 | 1030 | Shuangjiang |  | 61 | 1077 | Like tea shop |
| 26 | 1031 | 50 Lan |  | 62 | 1078 | Cocafado |
| 27 | 1032 | Yoursun Tea |  | 63 | 1079 | Nutsmilk |
| 28 | 1033 | MACU TEA |  | 64 | 1080 | Sugarcane mama |
| 29 | 1034 | Xing Fu Tang |  | 65 | 1081 | Woo Tea |
| 30 | 1035 | Dear Queen Tea |  | 66 | 1082 | Machi Machi |
| 31 | 1036 | XIAO CHA ZHAI |  | 67 | 1083 | Taro bubble |
| 32 | 1037 | ITSO |  | 68 | 1084 | Shakes Pure Tea |
| 33 | 1038 | I’MILKY |  | 69 | 1086 | Jin Fa Jia |
| 34 | 1039 | Chun Fun How |  | 70 | 1088 | Shian Ming |
| 35 | 1040 | ONEZO |  | 71 | 1089 | TrueWin |
| 36 | 1041 | Do It Yourself |  | 72 | 1090 | Tshing Tsuí Tê Phang |

**Supplementary Table S2.** Codebook: Labeling Information and Sugar Level Options among Brands.

| **Item** | **Coding** | |
| --- | --- | --- |
| **Brand Information** | | |
| Brand ID | Numbers | |
| Brand name | Recorded the brand name | |
| **Labeling Information** | | |
| Labeling acquisition | 1: obvious labeling  2: unobvious labeling  3: provided online after request | |
| Labeling presentation methods | 1: menu notes  2: at the order counter  3: poster  4: notice board  5: on the back of the menu  6: booklet  7: official website or online channels  8: no labeling or incomplete labeling | |
| **Sugar Levels Options** | |  |
| Options for sugar levels | Record the numbers of sugar levels options |  |
| Sugar level labeling | 0: no detailed information about sugar content  1: percentage  2: gram |  |
| Sugar level: more sugar | 0: no  1: yes | |
| Sugar level: regular-sugar | 0: no  1: yes | |
| Sugar level: 90% sugar | 0: no  1: yes | |
| Sugar level: less-sugar | 0: no  1: yes | |
| Sugar level: half-sugar | 0: no  1: yes | |
| Sugar level: low-sugar | 0: no  1: yes | |
| Sugar level: very low sugar | 0: no  1: yes | |

**Supplementary Table S2.** Codebook: Labeling Information and Sugar Level Options among Brands (continued).

| **Item** | **Coding** | |
| --- | --- | --- |
| Sugar level: sugar-free | 0: no  1: yes | |
| **Corresponding Sugar Contents of Common Sugar Levels** | |  |
| Sugar content: regular-sugar (g) | Record the regular-sugar content from the labeling or the regular-sugar contents of a large (L) size cup of black tea |  |
| Sugar content: less-sugar (g) | Record the less-sugar content from the labeling or calculate by sugar level percentage |  |
| Sugar content: half-sugar (g) | Record the half-sugar content from the labeling or calculate by sugar level percentage |  |
| Sugar content: low-sugar (g) | Record the low-sugar content from the labeling or calculate by sugar level percentage |  |

**Supplementary Table S3.** Codebook: Sugar and Energy Information for Hand-shaken Tea Drinks.

| **Item** | **Coding** | |
| --- | --- | --- |
| **Brand Information** | | |
| Brand ID | Numbers |  |
| Brand name | Recorded the brand name |  |
| **Beverage Information** | |  |
| Beverage name | Recorded the beverage name |  |
| Beverage category | 1: tea  2: tea with toppings  3: milk tea  4: milk tea with toppings  5: fruit tea  6: fruit tea with toppings |  |
| Portion size (mL) | Recorded the portion size from the labeling (mL) |  |
| Energy content (kcal) | Recorded the energy content from the labeling (kcal) |  |
| Sugar content: regular-sugar (g) | Recorded the regular-sugar content from the labeling (g) |  |
| Sugar content: half-sugar (g) | Recorded the sugar content of half-sugar by sugar level percentage |  |
| Sugar content: low-sugar (g) | Recorded the sugar content of low-sugar by sugar level percentage |  |
| **High Sugar Content Criteria** | | |
| The WHO recommendation: > 25 g/cup | 0: ≤ 25 g/L cup  1: > 25 g/L cup | |
| Warning Label criteria: > 5 g/100 mL | 0: ≤ 5 g/100 mL  1: > 5 g/100 mL | |
| Taiwan’s regulations for low-sugar  packaged beverages: > 2.5 g/100 mL | 0: ≤ 2.5 g/100 mL  1: > 2.5 g/100 mL | |

**Supplementary Table S4.** Codebook: Online Marketing for Hand-shaken Tea Drinks.

| **Item** | **Coding** |
| --- | --- |
| **Brand Information** | |
| Brand ID | Numbers |
| Brand name | Recorded the brand name |
| **Online Platforms** | |
| Official Facebook account | 0: no  1: yes |
| Official LINE account | 0: no  1: yes |
| Official Instagram account | 0: no  1: yes |
| Official brand website | 0: no  1: yes |
| Other online marketing platforms | 0: no  1: yes |
| Other online marketing platforms | Recorded the other online marketing channels |
| **Online Ordering Systems** | |
| Brand’s online ordering services | 0: no  1: yes |
| Food delivery platforms | 0: no  1: yes |
| **Information in Facebook Posts** | |
| Post ID | Numbers |
| Brands | 1: DaYung’s  2: CoCo  3: Presotea  4: MACU  5: TEATOP  6: TRUEWIN  7: KEBUKE |
| Post date | Recorded the post date |
| Like | Recorded the number of “likes” |
| Comments | Recorded the number of “comments” |
| Share | Recorded the numbers of “shares” |

**Supplementary Table S4.** Codebook: Online Marketing for Hand-shaken Tea Drinks (continued).

| **Item** | **Coding** |
| --- | --- |
| **Facebook Marketing Strategies** | |
| Co-branding | 0: no  1: yes |
| Special offers | 0: no  1: yes |
| Interactions with social media users | 0: no  1: yes |
| Specific beverage information | 0: no  1: yes |
| Cross-selling | 0: no  1: yes |
| Brand information | 0: no  1: yes |
| Nutrient and health marketing | 0: no  1: yes |

**Supplementary Table S5.** Distribution of Facebook Marketing Strategies.

| **Facebook Marketing Strategies** | ***N*** | **%** |
| --- | --- | --- |
| **Co-branding** | |  |
| Cross-industry cooperation | 117 | 20.9 |
| Children's roles/Cartoon characters | 23 | 4.1 |
| Celebrity endorsement | 16 | 2.9 |
| **Special Offers** | |  |
| Specific population/payment offer | 192 | 34.3 |
| Limited time offer | 187 | 33.4 |
| Specific store offer | 139 | 24.8 |
| Group buying offer | 134 | 23.9 |
| Single beverage offer | 78 | 13.9 |
| **Interactions with Social Media Users** | |  |
| Raffle | 68 | 12.1 |
| On-site interaction | 22 | 3.9 |
| “Comment” on the post | 17 | 3.0 |
| Tag friends/Write a post/Hashtag | 16 | 2.9 |
| “Like” the post/fan page | 9 | 1.6 |
| “Share” the post/fan page | 8 | 1.4 |
| Follow the fan page | 2 | 0.4 |
| **Specific Beverage Information** | |  |
| Beverage image | 460 | 82.1 |
| Beverage name | 393 | 70.2 |
| Promotion/Introduction of beverage ingredients | 221 | 39.5 |
| New beverage | 89 | 15.9 |
| Season limited/Region limited | 81 | 14.5 |
| URL of beverage-related information | 25 | 4.5 |
| **Cross-selling** | |  |
| Non-beverage-related product | 34 | 6.1 |
| Food (non-beverage) | 27 | 4.8 |
| Beverage-related product | 20 | 3.6 |

**Supplementary Table S5.** Distribution of Facebook Marketing Strategies (continued).

| **Facebook Marketing Strategies** | ***N*** | **%** |
| --- | --- | --- |
| **Brand information** | |  |
| URL of the official website or brand-related information | 388 | 69.3 |
| Holidays/Current affairs | 149 | 26.6 |
| Store announcement | 82 | 14.6 |
| Mascot | 64 | 11.4 |
| Upload cover photo/profile picture | 22 | 3.9 |
| **Nutrient and health marketing** |  |  |
| Containing fruit/juice | 284 | 50.7 |
| Fresh fruit and vegetable images | 188 | 33.6 |
| Fresh | 152 | 27.1 |
| Origin of raw ingredients | 107 | 19.1 |
| Seasonal ingredients | 97 | 17.3 |
| Milk | 84 | 15.0 |
| Original/Pure/Good tea | 59 | 10.5 |
| Tea leaves/teabags image | 40 | 7.1 |
| Natural/Organic/No additives | 25 | 4.5 |
| Other nutrient and health marketing  (Nourishing tonic, no caffeine, low calorie, etc.) | 13 | 2.3 |
| No sugar | 8 | 1.4 |
| No burden on health | 7 | 1.3 |
| Dietary fiber | 6 | 1.1 |
| Vitamin C | 5 | 0.9 |
| Nutrition/Health | 4 | 0.7 |
